# Navigating migration and cancer in Asia: A narrative analysis of stories told by Filipino migrant domestic workers with breast cancer

**DOI:** 10.1016/j.jmh.2025.100337

**Published:** 2025-05-29

**Authors:** Margo Turnbull, Xiaoyan I. Wu

**Affiliations:** Department of English and Communication, International Research Centre for the Advancement of Health Communication, Hong Kong Polytechnic University, Kowloon, Hong Kong, China

**Keywords:** Cancer, Identity, Migrant domestic workers, Hong Kong, Philippines

## Abstract

This article presents the narrative analysis of interview data collected from 15 migrant domestic workers (MDWs) from the Philippines who were diagnosed with breast cancer in Hong Kong. The analysis draws on a social constructionist understanding of identity as multiple and performed through language, communication, and social interaction to explore how these MDWs narrated their cancer experiences and changing identities as they worked to incorporate serious illness into their lives as MDWs. The narratives of these MDWs highlight their multiple and changing identities as they move and communicate across places, systems of migration and networks of relationships.

## Highlights

• Migration status is a key determinant of breast cancer outcomes for women in Asia.
• Migrant domestic workers navigate both employment and migration when seeking diagnosis.
• Recovery was facilitated or impeded by employers, families and social networks.
• Growth in migration highlights the need to understand more about diversity of cancer experiences.

## 1 Introduction: Migration, global health and breast cancer

The impact of changing patterns of migration on health is evident in the Asian regions which have significant levels of temporary intra-regional movement as many people move from low- and middle-income home countries to high-income host destinations for employment (Fong and Shibuya, 2020). In addition to highlighting the wealth inequality that drives this movement, these circular patterns of migration (Shivakoti, 2024) are increasingly linked to differences in health outcomes and the distribution of the burden of serious diseases like cancer (Bray et al., 2021; Soerjomataram and Bray, 2021; Bennett et al., 2020). Cancer *burden* refers to both incidence and mortality rates as well as the psychological, physical, social, human and economic ‘costs’ of the disease (Essue et al., 2020). Despite rapid technological advancements, the burden of cancer remains linked with geographical location (Hausman, 2019; Vineis and Wild, 2014) as people living in lower-income countries benefit the least from improvements in diagnosis and treatment (Molassiotis et al., 2019; Sharma, 2021). A significant body of literature has developed around the intersection of health, migration and cancer and it is widely accepted that migrants often have poorer outcomes in terms of recovery from cancer (Broom et al., 2020).

Although extant literature provides valuable insights into these issues, a majority of the underpinning research has been conducted in high-income countries – particularly in Australia, Europe, the UK and the US – with migrants who have re-located permanently to new host destinations (Broom et al., 2020; Burke et al., 2012; Goldstein et al., 2014; Kleinman et al., 1978; Scanlon et al., 2021; van Eechoud et al., 2016). Limited research has focused on the intersection of circular migration patterns and serious diseases like cancer in Asia. This article seeks to go some way to addressing this gap by extending prior exploratory research (Turnbull et al., 2024) to examine in detail the intersection of *temporary* migration systems and breast cancer. To do this, we use narrative analysis to explore Migrant Domestic Workers’ (MDWs’) experiences of breast cancer. The rationale for this focus is derived from the increases in incidence and prevalence levels of cancer in Asia as well as the need to situate studies of the disease within the broader sociopolitical contexts that facilitate certain types of migration. As argued elsewhere (Turnbull et al., 2024), temporary, transnational migrant workers are often in a unique position straddling two locations and thus decisions about health care are influenced by the structural factors that drive migration (employment, finances) as well as an awareness of disparities between home and host healthcare systems.

## 2 Context: Temporary migration and MDWs in Hong Kong

Systems for temporary migration for the purposes of employment are well established within the East and Southeast Asian regions (Fong and Shibuya, 2020). The Philippines has the most developed system for supporting its Overseas Foreign Workers (OFWs) (Piper, 2017) and this has been used as a model by other countries in Asia (Rodriguez, 2010). In the Philippines, the importance of individuals becoming OFWs is framed by the government as a way of displaying loyalty and devotion to families as well as being citizen ‘heroes’ helping the country (Parreñas, 2001). Between April and September 2021, 1.83 million Filipinos were registered as OFWs – 60 % of these workers were female (Philippine Statistics Authority 2022). The remittances OFWs send home to their families are a significant source of income for the Philippines. In 2023, $33.490 billion USD was sent back to the Philippines by OFWs (Philippine Statistics Authority 2024).

Hong Kong is a popular destination for Filipino MDWs and 199,516 MDWs were employed in the city in 2023 (Hong Kong Government 2024). The popularity of Hong Kong is influenced by its proximity to the Philippines and comparatively generous legal protections. Unlike other jurisdictions across the broader Asian regions, Hong Kong’s immigration and labour laws regulate minimum wages, rest days and the provision of health care to migrant workers (Hong Kong Immigration Department 2023). While MDWs are employed and have valid work visas, employers are legally obliged to ensure their healthcare needs are met and the contracts of MDWs cannot be terminated if they are on certified sick leave. Despite this legislation, issues with enforcement of laws, limited understanding of employment rights and the financial entanglements that often drive this circular migration increase the risk of exploitation of these women (Henderson, 2020; Ladegaard, 2023).

A significant body of literature has developed around MDWs in Hong Kong. There has been a particular focus on social networks (Piocos et al., 2022), health behaviours and health literacy (Bernadas and Jiang, 2016; Christie-de Jong and Reilly, 2020; Straiton et al., 2017; Turnbull et al., 2023), abuse and trauma (Ladegaard, 2023; Cheung et al., 2019) as well as the long-term effects of this (often unsuccessful) model of migrating out of poverty (Boersma, 2019; Spitzer et al., 2023). Despite this work, however, relatively little is known about the long-term health needs of these women as they age or if they are diagnosed with serious illnesses like breast cancer (Turnbull et al., 2024; Amrith, 2021). This lack of information is attributable to a variety of factors including the temporary nature of their employment - some women may only work in Hong Kong for one or two contracts while others stay for many years across non-continuous periods, often working into their 60s (Liao and Gan, 2020). Tracking disease amongst this population is also difficult - women who receive a diagnosis of cancer in Hong Kong are encouraged to register details with the local Philippine consulate, but it is up to individuals to do so.

This lack of data means that little is known about how MDWs diagnosed with breast cancer make decisions regarding the management of their treatment and recovery needs within the broader structures of their migration experiences. This is a significant gap in terms of tracking how female migrants recover from breast cancer and resume economic and social activity either in the host or home location. Given the lack of information and unique nature of the MDW population, a small-scale exploratory study led by the first author in 2022 examined the individual experiences of women with breast cancer (Turnbull et al., 2024). Findings of this study indicated that individual women’s decision-making about their healthcare needs was primarily influenced by their roles and identities as family financial providers and temporary migrants. The subsequent study reported in this article extended this work by examining how breast cancer experiences intersected with and were shaped by broader discourses and structures of migration in Asia. In addition to providing important insights into how temporary transnational migration shapes women’s health in Asia, it is of international relevance as a similar model of temporary migration is increasingly being used to address workforce shortages in high-income countries (Sweileh, 2024).

## 3 Methodology

This study adopted a qualitative research design to collect and analyse data in the form of narratives. Narrative analysis (De Fina, 2013; Fetzer, 2010; De Fina and Georgakopoulou, 2008) was used to trace expressions of and changes in identities through the stories participants told about their experiences of having breast cancer. Narratives offer valuable insights into how individuals think about, make sense of and incorporate events into broader biographies of their life. Importantly, narratives are often assembled through the interweaving of discourses about life and social issues that are of critical importance to the speaker and that are linked to broader social processes (De Fina, 2013). As argued by De Fina and King (2011), narratives can thus be seen to reflect and shape social realities and expressions of identity(ies). In this article, we use this approach to analysis to examine how narratives of migration and breast cancer are interwoven into the stories women tell about their lives and experiences.

### 3.1 Participant recruitment

The data reported in this article were collected as part of a larger multi-phase qualitative study examining health and disease amongst female MDWs led by the first author. Purposive sampling was used for participant recruitment, which was facilitated by the first author’s pre-existing contacts within the MDW community. Details of the research were shared with the organisers of an informal Filipino cancer support group in Hong Kong. These organisers then distributed this information to potential participants and coordinated interview times and locations. Eligible participants were women (a) from the Philippines who were legally employed as MDWs in Hong Kong; (b) aged over 18; and (c) diagnosed with cancer during their employment in the city. A total of 22 participants diagnosed with a variety of cancer types (e.g., breast cancer, bladder cancer and cervical cancer) were recruited. Narratives from 15 participants who were diagnosed with breast cancer were included in the analysis described in this article. Breast cancer was selected for this analysis due to its high incidence and prevalence rate in both Hong Kong and the Philippines.

### 3.2 Data collection and management

This research was granted ethical approval by the relevant institutional board (reference numbers: HSEARS20220317003–02). Data were collected through semi-structured interviews. The interview guide is included in Appendix A. Interviews were conducted in community settings. The interviewees were given the option of participating in English or Filipino and all chose to be interviewed in English. Field notes and observations made by the interviewer indicated that all participants were proficient English speakers and were able to engage in complex discussions about their experiences of having breast cancer in the language. Participants reported being taught English at school and English is the language used in the healthcare system by non-Chinese speakers in Hong Kong so these women were used to talking about breast cancer in English.

We acknowledge that interviews are sites for the negotiation of identities and the representation of social relationships (De Fina and King, 2011) and, hence, reflexivity and positionality of researchers in both data collection and analysis need to be acknowledged. All participants were interviewed by the first author (MT: Australian, white female). MT has extensive experience designing and conducting health communication research in Asia with multilingual colleagues and participants. MT’s previous exploratory research offered important insights into how to conduct this sensitive research with participants who may have concerns for their jobs and status in Hong Kong. The second author is an experienced qualitative researcher who has been involved in various communication projects in Hong Kong since 2017 with a particular focus on issues of migration and communication.

All interview sessions began with introductions and sharing of the information sheet and consent forms both verbally and in writing. Written information was provided in both English and Filipino. Principles of informed and voluntary consent were observed. Potential participants were encouraged to take time to read the information sheet and to ask questions about the research before opting to give consent to participate. It was emphasized that participation was voluntary and they could withdraw from the research at any time. All women approached about the research agreed to participate. Upon the completion of the interview, participants were given refreshments and a store voucher (valued at HKD $100, approximately USD $12). To ensure anonymity and confidentiality, no identifying details were collected or recorded during interviews and all data were anonymised and stored securely in password-protected files. Each participant was assigned a pseudonym, and these are used in this article.

All interviews were audio-recorded with permission from participants and transcribed verbatim by a student research assistant. Transcripts were checked for accuracy by the interviewer (MT). The interview recordings lasted for a total of 242 min (range 4 to 48 min), and combined transcripts were 35,611 words in length. Due to the research design (i.e., anonymity of participants) and the transient nature of some employment (i.e., key contacts reported that some interviewees had left Hong Kong by the end of the research project), transcripts of the interviews and the results of the analysis were not returned to participants for review or further discussion. Ways of addressing this potential limitation in future research are discussed in a later section of the article.

### 3.3 Data analysis

The narrative analysis presented here draws on the work of Georgakopoulou (2006), De Fina (2013) and Fetzer (2010), which foregrounds how identity(ies) are shaped and expressed in participant stories. Excel spreadsheets and Word documents were used for data storage and analysis. Both authors participated in data analysis, which began with repeated listening to the interview recordings, reviewing of interview transcripts, note taking and annotation. This individual work was then used in collaborative discussions to generate ideas for initial coding. The second author (XW) extracted and coded excerpts from the transcripts that reflected the participants’ experiences linked with expressions of identity. The authors then reviewed and discussed these to develop candidate themes. These were further coded and iteratively refined into final themes that reflected the interaction of cancer experiences and the structures and drivers of migration. The findings of the research are reported according to the consolidated criteria for reporting qualitative research [COREQ] (Tong et al., 2007).

## 4 Results

Narratives of 15 eligible MDWs were analysed. Their demographic information is summarised in Table A.

**Table A:** Participant demographic details (*N* = 15).

**Pseudonym**	**Age**	**Highest level of completed education**	**Marital status**
Emma	49	Secondary school	Separated
Ava	52	High school	Married
Anne	49	Vocational	Married
Mia	47	Secondary school	Married
Aria	54	Vocational	Married
Julie	53	High school	Separated
Tara	50	Secondary school	Married
Kate	59	Secondary school	Married
Dolly	47	College	Married
Ella	52	Secondary school	Single
Hazel	55	University	Married
Nina	50	High school	Married
Ruth	49	High school	Never married
Sandy	57	High school	Married
Celia	55	College	Married

*Note*. The original terms used by the participants to describe their education level and marital status were reported in the table even though there were some inconsistencies (e.g., vocational versus college).

Narrative analysis identified patterns in how these women experienced shifting identities as they processed their changing status from healthy women to ‘someone with breast cancer’. Their identity shifts and cancer experiences were framed by the structures of migration that included the factors that drove and sustained their migration to Hong Kong (i.e., financial need) and shaped the nature of their lives in the city. This is evident in the narration of their experiences of breast cancer and the ways in which they rationalised the ‘choices’ they made in terms of their transnational employment and relationships with employers and their families. Three themes were developed from their narratives (see Table B). Interview extracts are largely presented verbatim unless indicated with ellipsis (omission of words or sentences) or square brackets when words/grammar have been corrected for clarity.

**Table B:** Themes developed from the participants’ narratives.

**Themes**
1. Transitioning from being healthy to having breast cancer
2. Balancing new and old identities: Conflicting responsibilities and expectations
3. Systems and structures of transnational migration and breast cancer

### 4.1 Theme 1: Transitioning from being healthy to having breast cancer

All participants’ narratives about breast cancer began with describing how and when they noticed a change in their bodies. They carried out self-examination and most participants sought out medical screening immediately. These stories showed high levels of awareness of their own health and wellbeing.

Employers play a pivotal role in the participants’ cancer experiences and identity shifts, reflecting the characteristics of this unique form of migration. According to Hong Kong law, MDWs’ employers are legally obliged to ensure they receive adequate medical care and it is illegal to terminate the contract of a worker who is on certified sick leave. The individual employer thus takes on a key role in facilitating diagnosis and treatment. However, despite the laws in Hong Kong, there are issues with the enforcement and the disparities in knowledge and power between employers and employees complicate these relationships. This complexity is evident in how the participants recounted the involvement of their employer in the confirmation of the diagnosis and subsequent negotiation of treatment. Supportive employers were described by some women as providing emotional and financial support, keeping them company during their treatment at the hospital, encouraging them to be strong, reducing their workload, or reassuring them about the security of their employment. The following quotes exemplify what these women considered as supportive employers:

Ella: *After we take the result then my employer told me say so sad she’s crying for me … They treat me as a family already, even the kids, they crying, they say "Don’t worry. We’re family" … if during my treatment, if I’m cannot work, she do the housework. She’s very supportive.*

Dolly: *…luckily, we have a good employer. It supports us a lot because we have peace of mind that we don’t lost the visa while we are in department of oncologist or surgery department so it’s very essential for us to have a good employer.*

It was noteworthy that for some women, the shift in their role from helping their employer to being someone who was helped or cared for was a new dynamic for which they were grateful but it could also be a source of discomfort. This is illustrated in the following quote:

Ava: *They [the employers] are always supporting me, but then I usually help them to cook and then during that time, I’m so weak. They are the one who cook for me … I think that is not my, I’m not used to it, that someone will cook for me.*

Some participants reported a change in employers’ attitudes as time passed after the diagnosis and start of treatment. These changes were usually linked with threats to terminate the contract:

Ruth: *When I was diagnosed, they were like, "Oh, you don't, you don't go home to Philippines. You have to do your treatment here and we will fight this together" … This first week of August, I was just surprised that my boss called me and say, "Oh, our contract is finished." And then I was surprised because I know it's not finished. … But my boss insisted that our contract is finished and he wants me to leave his house like one week after that conversation.*

Other employers were described as unsupportive from the outset, refusing to pay for the treatment, stopping paying them properly, threatening to terminate their contract, or refusing to renew their contract. When undergoing treatment, some participants still had to work as usual even though they felt sick and weak from the treatment. Long working hours and heavy workloads affected individuals’ ability to cope with treatment and optimise their recovery. Several participants reported having to cancel or reschedule their treatment sessions or medical appointments because their employers would not release them from work. The role of unsupportive employers is illustrated in the following quotes.

Julie: *I almost sleep 1 o’clock, 2 o’clock in the morning, and then I wake up 7, like that, and then straight working. I should be finished my first cycle chemo but my employer lady not allow me that day so I have rebook my schedule … she always fighting me, want to terminate me.*

Nina: *They [the employers] asked me to do the things, but I told them I cannot carry heavy things, because my employer always asks me to do this one. Do this one. … I'm just recovering so I told them that I cannot carry heavy things … They don't care that if you are finish your surgery and chemotherapy, they don't care. They still ask you to do the things.*

The participants with unsupportive employers experienced tremendous stress or even depression because the features associated with the physical and psychological reality of having cancer conflicted with the requirements of their jobs. As cancer patients, they were entitled to attend treatment sessions and rest when necessary but their worry about being considered as troublesome and having their contracts terminated often drove them to put up with their employers and fulfil their expectations. These women’s financial needs and the access to healthcare in Hong Kong influenced some women to continue to insist on showing employers their physical ability to do their job, despite their diagnosis. This can be seen in the following extract:

Anne: *I don’t feel so much pain … I still can do my work. I only rest for one month, I have the sick leave certificate, one-month sick leave certificate. After that, I continue my work. Even though I’m on sick leave I still can do a simple work like dusting, sweep the floor, mop the floor.*

The participants needed to balance family financial needs with their health and some women had to tolerate abusive employers. For example, Aria described:

*I’m the only breadwinner of the family. I think of the family financially and I don’t want to be affected the support of my daughter. … [The employer did] not pay me, at the same time, the food, I sleep on the sofa, they don’t care even [when it’s] late. They make noise. They don’t care that your sleeping time. At the same time, if I go to the hospital they get mad because of the time, That’s why even I have examination or treatment checkup, sometime I do not go because of the situation of my boss is not very supportive … I was so depressed.*

Narratives told by the participants highlighted how the system of migration and their position as temporary migrants influenced the validation of their diagnosis and then shaped their ‘journey’ as a cancer patient. Their stories also illustrated the complexity of the relationship between employers and employees that is shaped by this particular type of migration. The key role of employers differentiates the experiences of these women from those of residents or permanent migrants.

### 4.2 Theme 2: Balancing new and old identities: Conflicting responsibilities and expectations

Our participants highlighted that their relationships with their families in the Philippines shaped their cancer experiences in fundamental ways. Family financial need was the most common and powerful driver of migration, and many women were the sole providers for their families. Importantly, many women had believed themselves to be physically healthy and thus they had to reconcile their own changed identity with their view of themselves and within the family in terms of being strong and able to provide financially. As Ruth highlighted, “*Acceptance is very important because if you don’t accept it, you cannot move forward*.”

Family members were a critical source of both emotional support and concern. Disclosing the diagnosis to families was difficult because they did not want them to worry and because often the women and their families had very negative beliefs about potential recovery from cancer. Therefore, sometimes they would choose to hide the diagnosis and only told their families when they had started to recover. Alternatively, others disclosed the diagnosis slowly and sought to constantly reassure their families throughout the process of disclosure. Thinking about the future of their children was identified as a motivating factor in their ‘fight’ against cancer so that they could recover and continue to be the breadwinners and support their children’s education and future.

In contrast, a few participants found it stressful when their families constantly messaged them about their diagnosis or treatment and indicated that the stress of the family exacerbated their own anxiety. Consequently, they would reduce the frequency of speaking to their families over the phone or video to avoid additional stress:

Ava: *Before I always talk with my family. I have video calls something like that but during that time that I’m diagnosed with cancer, I don’t want to talk to them. I don’t want to see them because every time I saw them, they cry so when they cry I cry, so when I start to cry I cannot stop, day night, day night, I cry, I cry, I cry.*

### 4.3 Theme 3: Systems and structures of transnational migration and breast cancer

As we discussed in the introduction to this article, there is significant inequality in access to oncology services across locations in the Asian regions. Temporary migrant workers are in the unique situation of being positioned across multiple healthcare systems and seeing differences in services and potential outcomes. All participants stated that they wanted to have their breast cancer treated in Hong Kong as they considered it to be more advanced and affordable than the services they could access in the Philippines. There were also additional financial supports available to interviewees in Hong Kong through the government Social Welfare Department and from non-government organisations. The following extracts illustrate the contrasts perceived by the interviewees.

Anne: *Even though I have I have this sickness but this is nothing because here [in Hong Kong] this is curable … If you’re in Philippines, the main problem is the medication and the hospital bill, the money, so since I’m here in Hong Kong everything is paid by the employer and there’s nothing to pay, I think HK$100, $85 for the medicine, something is affordable here in Hong Kong. … We don’t think that cancer is curable … Scary, cancer is so scary.*

Ruth: *… the connotation of cancer in Philippines is that when you have cancer, it's you're, you're done. You're finished. Unlike here, you know, you still have, you know, a lot of hope. In Philippines, it's you're, you're finished. That's your death sentence.*

Julie: *They’re very kind, especially all my doctors and the social welfare, so I was Thanks God every time I have the scan of my body, I don’t have to pay – it’s free. Because in Philippines, every time you go hospital it’s have to have money. They will not touch you without money.*

These extracts highlight the unique transnational position that these MDWs occupy. Although they are in low-skilled and relatively low-paid jobs in Hong Kong, they have access to healthcare that would be unavailable to them in the Philippines. In this sense, migration opens up new opportunities for their health outcomes, which accounts for their tendency to prioritise their ongoing employment. This lends further urgency in terms of the negotiation of their employment status and maintenance of their work visas.

The Filipino MDW community in Hong Kong is well established and its social networks are vital sources of support and information. It is also a key component of the broader macro system of migration which includes government stakeholders such as the Hong Kong Labour Department and the Philippines Consulate. The women in our research narrated how their cancer experiences involved negotiation with these groups and structures as they often sought to assert their rights and gather information. For example, several participants with unsupportive employers described their cancer experiences as involving interaction with their employers, the Philippine Consulate and the Hong Kong Labour Department. The experiences of Nina and Ruth exemplify this. Initially, the employers of both women wanted to terminate their contracts after they were diagnosed with breast cancer. Nina and Ruth had to advocate for themselves and their rights as employees by seeking out help and advice from various sources. The following extract illustrates the extra work these women had to do to access treatment in Hong Kong.

Nina: *I call the Philippine Consulate when they when they asked me about my situation, they asked me to call another number. The office for the welfare of Filipinos. It's OWWA.*^1^ *… When I call the OWWA, they told me, it seems like they blaming me for having a cancer … OWWA said, “I think you better call (the Consulate) back again” … They [OWWA] said to me, because if you come, if you come here, you will see all other domestic workers that having a problem, and then you will talk to them, you will get stressed. … I get so mad.*

After Ruth’s employer made it clear that they wanted to illegally terminate her contract, Ruth also sought help from the Philippine Consulate, which turned out to be a negative experience:

Ruth: *They were really useless. I didn't get even any answer to any of my questions. I didn't get any help … They even told me to just go home because it's better to go home, for me to have to spend some time to my family. It's like saying that, oh, your time is, you know, you have to go home because you have to spend time for your family … I told her that, oh, are you telling that I'm going to die soon?*

Ruth subsequently went to the Hong Kong Labour Department and was advised that her rights were protected by law and that she could make a complaint about the employer if she wanted to. Ruth, however, decided to negotiate with the employer who offered a financial settlement and agreed not to cancel Ruth’s work visa in return for her leaving the house. Ruth described leaving the house late at night and walking around the city looking for a place to stay.

Ruth: *I went [from] my boss house around 10:00 pm and so the mission is closed. So I contacted my I have a friend who has an apartment, and I ask her to if I can stay there for a night. And then she said, yeah, yeah, you can just come over here.*

These extracts illustrate the work Ruth and Nina had to do to negotiate and validate their identities as newly diagnosed breast cancer patients, which also reflected the experiences of other participants whose employers insisted on the premature termination of their contract. This work involved navigating their relationship with their employers, seeking help from government institutions and advocating for their own rights. This work took energy and was driven, in large part, by the contrast they perceived between the oncology services in the Philippines and in Hong Kong.

In addition to seeking out support from government agencies, all participants detailed the valuable support provided to them by community groups. These groups were run by other MDWs who had shared experiences and insider knowledge about their challenges and struggles. They considered the group involved in this project a significant source of emotional support. The shared experiences as MDWs with cancer allowed for mutual understanding and contributed to a sense of belonging. Being with this group reduced their fear towards cancer and the uncertainties associated with the prognosis ahead of them and alleviated their sense of helplessness and loneliness. The following extracts illustrate the benefits of this support.

Ava: *[The group] is a very big support emotion because at the time that I found out I have a cancer, I don’t want to talk to anybody … I think for 2, no, 3 months, I go alone, go with the doctor because my employer cannot go with me so I go alone … So when [I] find out this group so we talk, I’m so happy because I release everything and then I can all the question in my mind is all clear and then emotion because everybody know.*

Aria: *When I did not meet this community, oh my goodness, I don’t know what to – sometimes I want to die that time. I’m not kidding because I was lost, my memory cause, you know, the pain, the depression, I was I want to fight but … too much whys, ifs questions on my mind, very hard from, away from the family to have the treatments and if you just sickness like cancer.*

## 5 Discussion

Migration status has been clearly linked with access to oncology services and outcomes of cancer treatment. However, much of the literature exploring these links has drawn on research with migrants who have migrated permanently to a new country. Permanent migrants face a range of challenges in terms of their health that cluster around issues of acculturation and linguistic and cultural diversity in their new countries (Lockhart et al., 2020). The research in this article focused on a particular subpopulation of migrants – that is, MDWs who engage in repeated cycles of temporary or circular migration in Asia. These women occupy a unique position across home and host countries - despite years of working in locations like Hong Kong, MDWs do not accrue residency rights but do have access to many public and health services as long as they retain their work visas. The participants in our research described developing a keen awareness of the differences in health systems across Hong Kong and the Philippines and the potential impact of this on their long-term recovery from breast cancer. This, in turn, influenced the employment and treatment decisions these women made and how they developed their emergent identities after their diagnosis.

In this article, we have extended the findings of previous related work (Turnbull et al., 2024) to make broader connections with how women constructed their identities within the affordances and constraints of the migration system. Our findings highlight the emotional stress these women experience in the process of seeking out a medical diagnosis and accepting their new identity as a breast cancer patient and their reliance on their employer. This reliance meant that if employers were emotionally and financially supportive, the women believed they were likely to have positive outcomes from their treatment in Hong Kong. Other women described anxiety and fear linked with unsupportive employers and detailed how and why they often struggled to meet impossible expectations and tolerated abuse in order to keep their jobs and access to treatment. It is essential to note that these actions and decisions were made at the level of individuals, but these people were situated within a broader migration system assembled across home and host locations that directs and limits the options of many women. As argued elsewhere [(Ladegaard, 2015), p. 214], the notion of “choiceless choice” for some MDW workers in exploitative or abusive situations may be manifest in inaction or tolerance of the intolerable.

In our research with MDWs with breast cancer, we identified distinct patterns in terms of how they assembled their changed identities after being diagnosed with breast cancer. Trusson et al. (2016) referred to the legitimation of the cancer patient through diagnosis and then subsequent transition to a socially sanctioned ‘sick’ role. The MDWs in this research had vastly different experiences in terms of their identity and transition to ‘becoming’ a person with breast cancer. These experiences were heavily influenced by their status as temporary, transnational migrants and were shaped by their dependence on individual employers. These additional layers of identity work present in our narratives contrast with other studies of cancer amongst permanent migrants. It is also of note that this system of migration places employers in difficult positions as they become carers for MDWs and as such take on highly emotional roles that in other situations may be filled by partners, spouses or close family members. The migration system in Hong Kong provides advice for dispute resolution between employers and MDWs but does not offer support or training in how to deal with health crises.

Findings of our analysis converged with others in terms of the vital role of social support systems and networks for MDWs’ health [e.g., (Anjara et al., 2017; Chung and Mak, 2020)]. For some participants in our research, as well as in other studies (Chung and Mak, 2020), having daily contact with friends in Hong Kong reduced stress and improved their mental health but having daily or regular contact with family members back home did not necessarily have such positive effects. Specifically, being a member of a community with shared experiences (e.g., migration status, breast cancer) can facilitate their identity work by providing emotional support, reassurance and hope. However, communication with family members in the Philippines may sometimes exacerbate levels of anxiety and stress. It is also increasingly acknowledged that the dominant discourses in some countries that encourage low-income women to engage in this circular migration (Parreñas, 2001) impact family dynamics and increase psychological isolation in times of health or personal crisis.

Our research into the experiences of MDWs with breast cancer highlights the need to understand more about the diverse types of migration and links with health and disease across the lifespan. Circular migration is becoming increasingly popular at a global level as more people move on a temporary basis from lower to higher-income destinations. This has multiple effects on individuals as many people experience the negative impacts of fragmented healthcare as well as become aware of the disparities in healthcare systems across geographical locations. Lugosi et al. (2023) discuss systems in terms of ecologies that include a variety of stakeholders (individuals, families, government, non-government entities etc.) that shape experiences and determine the trajectories of individual migrants. Given the growth in movement and access to information, integrating individual experiences into the critique of macro ecologies will provide insights into how to work to address the underlying and crucial factors that shape health and disease for migrants and their families.

There are three limitations associated with this study that can be addressed in future work. Firstly, this research focused only on MDWs from the Philippines who often have comparatively high levels of education and strong social support systems in Hong Kong (Piocos et al., 2022). Workers from other countries of origin (e.g., Indonesia, Thailand) may have different levels of general and health literacy and social supports that will affect their experiences of breast cancer. Secondly, interview transcripts and findings were not discussed with interviewees. This was influenced by confidentiality (i.e. the interviewer did not have contact details for most participants) and limited access to participants. Future research designs would be strengthened by the inclusion of creative opportunities for participant feedback and multilingual engagement (this is particularly important for people with lower levels of English proficiency). Finally, this research did not include employers of MDWs. Given the nature of the employment relationship, their legal obligations and the challenges of providing care for someone with a serious illness, including their perspectives would be valuable.

## 6 Conclusion

The analysis discussed in this article contributes novel insights into the identities of a unique group of migrant women actively navigating breast cancer in Hong Kong. The narratives of these MDWs highlight their multiple and changing identities as they move and communicate across places (the Philippines and Hong Kong), networks of relationships (as mothers, wives, income earners, employees, migrants, and patients) and work to incorporate breast cancer and its treatment in their bodies and lives. The research described in this article draws attention to the diversity of migration and the need to consider the range of experiences that migrants have. In addition to focusing on a unique and often hidden or overlooked subpopulation of women, this research underscores the complexity of the psychosocial dimensions of migration, breast cancer, and global health inequality.

## CRediT authorship contribution statement

**Margo Turnbull:** Writing – original draft, Validation, Supervision, Resources, Project administration, Methodology, Investigation, Funding acquisition, Formal analysis, Data curation, Conceptualization. **Xiaoyan I. Wu:** Writing – original draft, Validation, Project administration, Methodology, Investigation, Formal analysis, Data curation.

## Declaration of competing interest

The authors declare that they have no known competing financial interests or personal relationships that could have appeared to influence the work reported in this paper.

## Appendix A: Interview guides

1. Tell me the story of your cancer. (Prompt questions: When did you first think or feel that something was wrong or different? Did you talk to someone? Who? Where? When?)
2. How has cancer affected your life and work here in Hong Kong?
3. How did your employer respond to the news of your diagnosis of cancer?
  *(Prompt questions: Describe your relationship with your employer before your diagnosis and treatment. Has your relationship with your employer changed since your diagnosis? If so, in what ways?)*
4. Do you think that cancer has affected your family in the Philippines? How?
5. How do you see your future?
6. What are your plans?

## Footnotes

1 OWWA refers to the Overseas Worker Welfare Administration, which is a Philippine government agency that provides support to its Overseas Foreign Workers and their families.
